# Long‐Term Trends and Projections of the Global Epilepsy Burden: Insights From the Global Burden of Disease Study 2021

**DOI:** 10.1002/hcs2.70081

**Published:** 2026-05-26

**Authors:** Du Cai, Xianze Li, Qiwen Yuan, Zhongming Lian, Chenyu Zhao, Baotian Zhao, Xiu Wang, Chao Zhang, Lin Sang, Wenhan Hu, Xiaoqiu Shao, Jianguo Zhang, Shichuo Li, Jiajie Mo, Kai Zhang

**Affiliations:** ^1^ Department of Neurosurgery, Beijing Tiantan Hospital Capital Medical University Beijing China; ^2^ Department of Anesthesiology The Third Affiliated Hospital of Zhengzhou University Zhengzhou China; ^3^ School of Pharmacy Queen's University Belfast Belfast United Kingdom; ^4^ Department of Neurosurgery Beijing Fengtai Hospital Beijing China; ^5^ Department of Neurosurgery, Beijing Neurosurgical Institute Capital Medical University Beijing China; ^6^ Department of Neurology, Beijing Tiantan Hospital Capital Medical University Beijing China; ^7^ China Association Against Epilepsy Beijing China

**Keywords:** disease burden, epilepsy, Global Burden of Disease 2021, projections

## Abstract

**Background:**

Epilepsy is a common neurological disorder imposing significant global health burdens. A comprehensive understanding of its temporal and spatial trends is critical for informing policies and guiding interventions.

**Methods:**

This study assessed the burden of epilepsy from 1990 to 2021 worldwide, in Asia, and in China, and projected trends to 2050. We used data from the Global Burden of Disease 2021 study to evaluate the incidence, prevalence, mortality, and disability‐adjusted life years (DALYs) related to epilepsy. Joinpoint regression, age‐period‐cohort analysis, and autoregressive integrated moving average forecasting were used to examine temporal trends and project future patterns. We also assessed the influence of socioeconomic development, health service coverage, and risk factors on the epilepsy burden, focusing on regional disparities.

**Results:**

From 1990 to 2021, the incidence and prevalence of epilepsy showed a significant upward trend, while mortality and DALY rates showed a significant downward trend. China exhibited sharper reductions in mortality (average annual percent change = −2.64) and DALY rates (average annual percent change = −1.79). Inequalities persist, with low socio‐demographic index countries showing a disproportionately higher epilepsy burden. In China, improved universal health coverage and socioeconomic development are correlated with a decreasing burden. Alcohol use remains a key modifiable risk factor, particularly among men. Projections suggest a continued shift in the epilepsy burden toward older adults, with those aged ≥ 70 years contributing the largest share of future cases.

**Conclusions:**

Epilepsy remains a major global health concern with a substantial and uneven disease burden. Comprehensive burden analyses are essential for guiding public health policy, optimizing resource allocation, and developing targeted prevention and intervention strategies.

## Graphical abstract

Targeted interventions addressing socioeconomic inequalities and health service disparities are essential for mitigating future epilepsy burdens, especially in low‐resource settings.

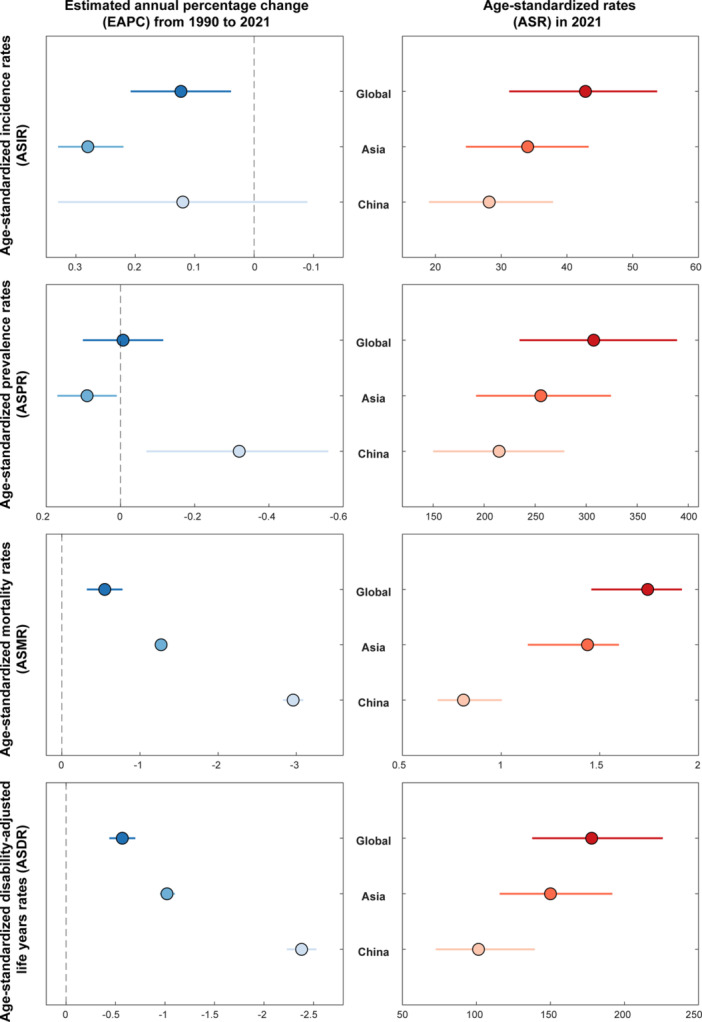

**Abbreviations**
AAPC: average annual percent change
ADF: Augmented Dickey–Fuller
APC: age‐period‐cohort
ARIMA: autoregressive integrated moving average
ASDR: age‐standardized DALYs rate
ASIR: age‐standardized incidence rate
ASMR: age‐standardized mortality rate
ASPR: age‐standardized prevalence rate
ASR: age‐standardized rate
CI: confidence interval
DALYs: disability‐adjusted life years
EAPC: estimated annual percentage change
GBD: Global Burden of Disease
GNI: gross national income
HDI: Human Development Index
IGAP: Intersectoral Global Action Plan
LEB: life expectancy at birth
MYS: mean years of schooling
NCD: non‐communicable disease
RMNCH: reproductive, maternal, newborn, and child health
SDI: Socio‐demographic Index
SII: slope index of inequality
UHC: Universal Health Coverage
UI: uncertainty interval
WHO: World Health Organization

## 1 Background

Epilepsy, which affects approximately 65 million people worldwide, remains a significant global public health challenge. People with epilepsy experience a three‐fold increased risk of premature death, imposing substantial burdens on families and healthcare systems worldwide [1, 2]. With rising global life expectancy and improved survival from neurological insults such as stroke and traumatic brain injury, the global epilepsy population is expected to increase significantly in the coming decades [3]. Consequently, comprehensive disease burden assessments and accurate projections of epilepsy are essential for targeted resource allocation and healthcare planning [4].

The World Health Organization (WHO) has identified epilepsy as a significant public health challenge, particularly in low‐ and middle‐income countries where the majority of epilepsy cases reside. The WHO's Intersectoral Global Action Plan (IGAP) on Epilepsy and Other Neurological Disorders 2022–2031 underscores the urgent need for coordinated efforts to improve diagnosis, treatment, and care for epilepsy, while addressing the substantial treatment gap and stigma associated with the disorder [5]. Current epidemiological studies using Global Burden of Disease (GBD) data have substantially advanced our understanding of the global patterns of epilepsy. While these studies provide valuable insights, prior analyses have primarily focused on descriptive trends or specific subtypes of epilepsy across broad regions, with limited attention to detailed, multidimensional assessments and long‐term forecasts, especially within rapidly evolving regions like Asia and China [6, 7, 8]. Given that approximately 80% of epilepsy cases reside in low‐ and middle‐income countries, predominantly within Asia, addressing regional disparities and socioeconomic influences through precise epidemiological projections is crucial for reducing the substantial treatment gaps [9].

Despite these efforts, significant gaps remain in our understanding of the long‐term trends and future projections of epilepsy burden, particularly in regions undergoing rapid demographic and socioeconomic changes. This study, with a particular focus on Asia and China, systematically analyzed trends and disparities in the global epilepsy burden from 1990 to 2021, while providing robust projections to 2050. Leveraging the GBD 2021 dataset, we employed advanced statistical methodologies to elucidate temporal‐spatial trends, predict future burdens, and integrate socioeconomic and healthcare accessibility factors to better understand the relationship between epilepsy burden and developmental contexts. Our study innovates by being the first to integrate Joinpoint regression, age‐period‐cohort (APC) analysis, and autoregressive integrated moving average (ARIMA) modeling, providing a comprehensive comparison of epilepsy burden trends across global, Asian, and Chinese contexts. By incorporating socioeconomic factors and health service coverage into our analysis framework, we fill the gap in regional precision forecasting and quantification of inequalities. By addressing these gaps, our study aims to provide actionable insights for policymakers and healthcare planners to develop targeted interventions and optimize resource allocation, ultimately improving health outcomes in regions disproportionately affected by epilepsy [10, 11, 12].

## 2 Methods

### 2.1 Data Source

The GBD 2021 dataset (https://vizhub.healthdata.org/gbd-results/) encompasses 204 countries and territories and provides estimates for 371 diseases and injuries and 88 risk factors from 1990 to 2021 [13]. All data used are available for public use and do not require further ethical approval. The incidence, prevalence, mortality, and disability‐adjusted life years (DALYs) of epilepsy with the corresponding 95% uncertainty interval (UI) were obtained from the GBD 2021 (Supporting Information). The GBD study determines the distribution of estimates by considering sampling error in the input data, uncertainty in model coefficients, severity distribution, and residual weight uncertainty. The 95% UIs are ultimately defined based on the 2.5th and 97.5th percentiles of 1000 draws from the posterior distribution centered on the mean estimate. DALYs, which represent the sum of years of life lost due to premature mortality and years lived with disability, were used as the primary measure of disease burden, providing an integrated view of the impact of epilepsy on population health.

### 2.2 Average Annual Percent Change (AAPC)

To assess the AAPC, the Joinpoint regression model was used to analyze the trend of age‐standardized incidence rates (ASIR), age‐standardized prevalence rates (ASPR), age‐standardized mortality rates (ASMR), and age‐standardized DALY rates (ASDR) over time. The basic principle of the regression is to divide the trend in the time series into several segments by identifying the Joinpoint of the model, including incidence, prevalence, mortality, and DALY rates, which identifies the position and number of Joinpoints in the model (Supporting Information) [8].

### 2.3 Predictive Estimation of Trend on Epilepsy Burden

This study employed the estimated annual percentage change (EAPC) to assess and predict trends in the epilepsy burden over the study period (1990–2021), including the ASIR, ASPR, ASMR, and ASDR. Positive EAPC values indicate an increasing trend, whereas negative values indicate a decreasing trend. If the 95% confidence interval (CI) of the EAPC crossed zero, the trend was considered stable. This approach allowed for a precise evaluation of how the epilepsy burden has evolved and facilitated robust projections of future trends up to 2050 (Supporting Information).

### 2.4 Cross‐National Inequality Analysis

The epilepsy burden and its relationship with socioeconomic conditions were assessed using the Socio‐demographic Index (SDI), a composite measure ranging from 0 (lowest development) to 1 (highest development). The SDI captures variations in income, education, and fertility rates, reflecting the overall social and economic development in each region. Countries and regions were categorized by SDI to evaluate disparities in the epilepsy burden [14]. Health inequalities were further analyzed using two complementary metrics: the slope index of inequality (SII), and the concentration index. The SII quantifies absolute inequality by measuring the difference in epilepsy burden between populations with the highest and lowest SDI values, where positive values indicate a concentration of burden in high‐SDI regions and negative values indicate a concentration in low‐SDI regions. In contrast, the concentration index measures relative inequality, assessing how the epilepsy burden is distributed across the full range of SDI, with positive values indicating a disproportionate burden in high‐SDI regions and negative values reflecting a concentration in low‐SDI regions. Together, the SII and concentration index provide insights into both absolute and relative disparities in the epilepsy burden.

### 2.5 Measurement of Socioeconomic Development

Beyond SDI, the relationship between epilepsy burden and broader socioeconomic development was further explored using four key indicators: Human Development Index (HDI), reflecting a country's average achievements in health (life expectancy at birth, LEB), education (mean years of schooling), and standard of living (gross national income [GNI] per capita), indicating the average lifespan under current mortality rates; GNI per capita, representing the total income of a country's residents adjusted for purchasing power parity; and mean years of schooling (MYS), capturing the average education level of adults aged 25 and above. These indicators provide a comprehensive framework for assessing how socioeconomic conditions influence the epilepsy burden globally, in Asia, and in China.

### 2.6 Measurement of Health Service Coverage

The relationship between epilepsy burden and health service coverage was assessed using four key indicators: Universal Health Coverage (UHC), which reflects the availability and accessibility of essential health services; reproductive, maternal, newborn, and child health (RMNCH), which measures the coverage of critical maternal and child health services; service capacity; and non‐communicable diseases (NCDs) coverage, which assesses health system capacity for the prevention and management of chronic diseases. These indicators were used to evaluate how variations in health service coverage influenced the epilepsy burden globally, in Asia, and in China.

### 2.7 Risk Factors

In this study, we assessed the impact of alcohol use on epilepsy burden using data from the GBD 2021 Comparative Risk Assessment framework. Alcohol use was selected as a representative modifiable behavioral risk factor, given its well‐documented association with epilepsy‐related mortality, particularly among men. This focused approach enabled us to explore temporal trends and regional disparities in alcohol‐attributable epilepsy burden across globally, Asian, and Chinese populations.

### 2.8 APC Model

The APC model was applied to quantify how the epilepsy burden has evolved across different age groups (biological susceptibility), historical periods (temporal factors), and birth cohorts (differences across generations), providing a comprehensive understanding of temporal and generational dynamics [15]. The model results were reported as risk ratios, reflecting the relative burden of epilepsy for each period and cohort compared to the reference categories. This approach enabled a nuanced understanding of how the epilepsy burden is influenced by demographic and temporal factors, offering insights into age‐specific prevention and control strategies (Supporting Information).

### 2.9 ARIMA Model

To generate robust projections of epilepsy burden, the ARIMA model was applied to predict trends in the ASIR, ASPR, ASMR, and ASDR of epilepsy across multiple age groups (< 20, 20–24, 25–29, 30–34, 35–39, 40–44, 45–49, 50–54, 55–59, 60–64, 65–69, 70–74, 75–79, and 80+ years). Before model fitting, stationarity of the time series was assessed using the Augmented Dickey–Fuller (ADF) test. The ADF test evaluates the null hypothesis that a unit root is present in the time series, implying non‐stationarity. If the *p*‐value of the ADF test is less than 0.05, the null hypothesis is rejected, and the time series is deemed stationary. For time series that failed the stationarity test, differencing (*d*) was applied iteratively until stationarity was achieved. The ARIMA model order, characterized by the autoregressive term (*p*), differencing order (*d*), and moving average term (*q*), was determined using the auto.arima() function, which leverages the Akaike Information Criterion to identify the model that best balances goodness‐of‐fit with model complexity [16, 17]. ARIMA is a widely used time‐series forecasting method that accounts for historical trends, seasonal patterns, and random fluctuations in data, making it particularly suitable for predicting future trends in health metrics [12].

## 3 Results

### 3.1 Global, Asian, and Chinese Trends of Epilepsy Burden From 1990 to 2021

From 1990 to 2021, the incidence and prevalence of epilepsy exhibited fluctuating trends globally, in Asia, and in China. Globally, the incidence showed a modest increase over the entire period, with an AAPC of 0.37 (95% CI: 0.35, 0.39), while the fastest rise occurred between 1990 and 1999, with an AAPC of 0.73 (95% CI: 0.68, 0.77). The prevalence also slightly increased with AAPC at 0.21 (95% CI: 0.20, 0.23), although with a recent plateau. Conversely, the mortality and DALY rates consistently declined, with the AAPC for mortality and DALY rates being −0.55 (95% CI: −0.66, −0.44) and −0.88 (95% CI: −0.98, −0.77), respectively. Within Asia, the incidence and prevalence increased more rapidly, especially during the early period (1990–1999), with an AAPC of 1.01 (95% CI: 0.91, 1.11) for incidence and 1.13 (95% CI: 1.03, 1.22) for prevalence. In China, after a significant initial rise, the AAPC was 2.57 (95% CI: 2.43, 2.70) for incidence and 2.26 (95% CI: 2.14, 2.38) for prevalence from 1990 to 1999. Both metrics decreased notably between 2000 and 2010, followed by a moderate rebound thereafter. Notably, China exhibited the sharpest decline in epilepsy‐related mortality and DALY rate, with an AAPC of −2.64 (95% CI: −2.84, −2.44) and −1.79 (95% CI: −1.96, −1.62) from 1990 to 2021, respectively, indicating substantial improvements in managing the epilepsy burden (Table 1).

**Table 1:** The incidence, prevalence, mortality, and DALY rates of epilepsy and its AAPC globally, in Asia, and in China from 1990 to 2021.

		Incidence		Prevalence		Mortality		DALYs	
Region	Year	AAPC (95% CI)	*p*	AAPC (95% CI)	*p*	AAPC (95% CI)	*p*	AAPC (95% CI)	*p*
Global	1990–1999	0.73 (0.68, 0.77)	< 0.001	0.73 (0.70, 0.76)	< 0.001	−0.39 (−0.51, −0.26)	< 0.001	−0.17 (−0.24, −0.11)	< 0.001
	2000–2010	0.19 (0.17, 0.20)	< 0.001	0.02 (0.02, 0.03)	< 0.001	−0.84 (−1.02, −0.67)	< 0.001	−0.79 (−0.86, −0.73)	< 0.001
	2011–2021	0.25 (0.23, 0.27)	< 0.001	−0.01 (−0.04, 0.02)	0.60	−0.29 (−0.43, −0.14)	< 0.001	−0.51 (−0.68, −0.35)	< 0.001
	1990–2021	0.37 (0.35, 0.39)	< 0.001	0.21 (0.20, 0.23)	< 0.001	−0.55 (−0.66, −0.44)	< 0.001	−0.88 (−0.98, −0.77)	< 0.001
Asia	1990–1999	1.01 (0.91, 1.11)	< 0.001	1.13 (1.03, 1.22)	< 0.001	−0.66 (−0.90, −0.42)	< 0.001	−0.15 (−0.25, −0.04)	0.007
	2000–2010	0.14 (0.12, 0.15)	< 0.001	0.03 (0.02, 0.05)	< 0.001	−1.60 (−1.91, −1.29)	< 0.001	−1.34 (−1.47, −1.22)	< 0.001
	2011–2021	0.35 (0.32, 0.38)	< 0.001	0.04 (0.01, 0.07)	0.01	−1.15 (−1.40, −0.90)	< 0.001	−1.04 (−1.33, −0.75)	< 0.001
	1990–2021	0.46 (0.44, 0.48)	< 0.001	0.34 (0.33, 0.36)	< 0.001	−1.21 (−1.31, −1.10)	< 0.001	−0.52 (−0.58, −0.47)	< 0.001
China	1990–1999	2.57 (2.43, 2.70)	< 0.001	2.26 (2.14, 2.38)	< 0.001	−2.40 (−2.73, −2.08)	< 0.001	−0.84 (−0.95, −0.73)	< 0.001
	2000–2010	−0.54 (−0.64, −0.45)	< 0.001	−0.95 (−1.05, −0.85)	< 0.001	−3.27 (−3.65, −2.88)	< 0.001	−2.98 (−3.35, −2.61)	< 0.001
	2011–2021	0.65 (0.37, 0.94)	< 0.001	0.36 (−0.09, 0.81)	0.11	−1.99 (−2.28, −1.70)	< 0.001	−1.09 (−1.47, −0.71)	< 0.001
	1990–2021	0.78 (0.65, 0.91)	< 0.001	0.43 (0.28, 0.59)	< 0.001	−2.64 (−2.84, −2.44)	< 0.001	−1.79 (−1.96, −1.62)	< 0.001

Abbreviations: 95% CI, 95% confidence interval; AAPC, average annual percent change; DALY, disability‐adjusted life year.

**Figure 1:**
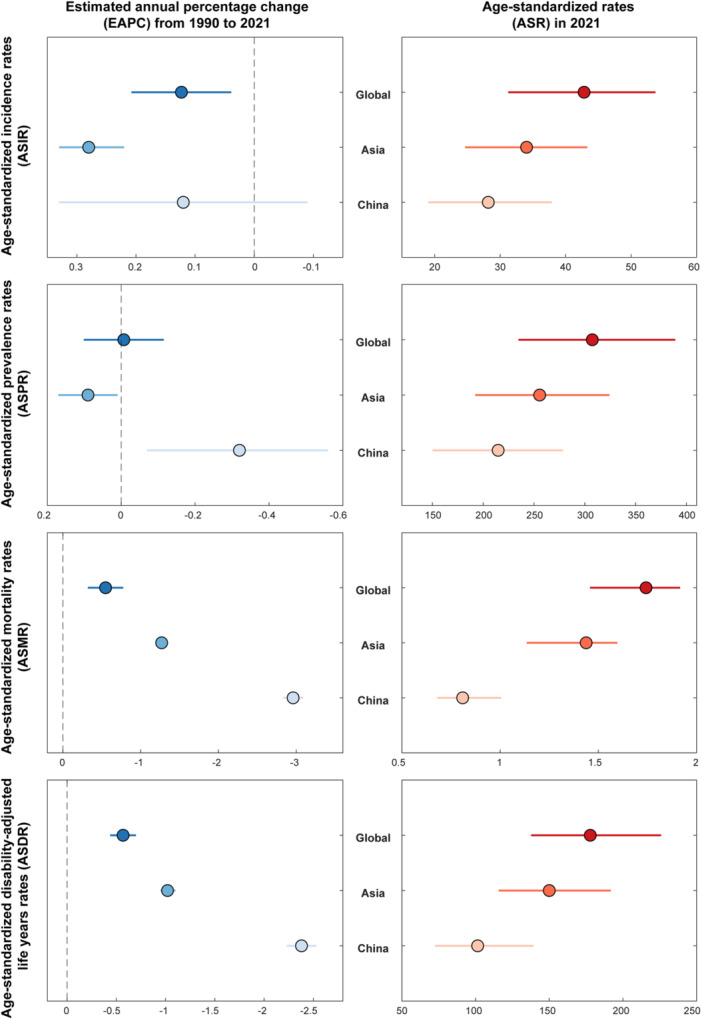
Global, Asian, and China‑specific distributions and temporal trends in the age‑standardized burden of epilepsy. Forest plots display the mean values and 95% CIs of the EAPC (left panels) from 1990 to 2021 and the ASRs (right panels) in 2021. Panels correspond to ASIR, ASPR, ASMR, and ASDR. ASDR, age‑standardized disability‑adjusted life‑year rates; ASIR, age‑standardized incidence rate; ASMR, age‑standardized mortality rate; ASPR, age‑standardized prevalence rate; ASR, age‑standardized rate; CI, confidence interval; EAPC, estimated annual percentage change.

In 2021, there were significant differences in the distribution of the epilepsy burden, which exhibited distinct regional variations globally, in Asia, and in China. Global ASIR, ASPR, ASMR, and ASDR of epilepsy in 2021 were 42.82 (95% UI: 31.23, 53.72), 307.37 (95% UI: 234.70, 389.02), 1.74 (95% UI: 1.46, 1.92), and 177.84 (95% UI: 137.66, 225.90), respectively. The epilepsy burden of the ASIR and ASPR was concentrated in countries such as Europe, Africa, and Latin America. In 2021, the ASIR and ASPR in Ecuador were 94.94 (95% UI: 29.94, 160.51) and 710.80 (95% UI: 226.24, 1140.83), respectively. However, the epilepsy burden of ASMR and ASDR was concentrated in African and Southeast Asian countries. Among them, Zambia's ASMR and ASDR in 2021 were 12.94 (95% UI: 9.47, 17.08) and 746.45 (95% UI: 505.83, 1031.36), respectively. The ASIR, ASPR, ASMR, and ASDR in Asia were 34.03 (95% UI: 24.64, 43.31), 255.57 (95% UI: 192.16, 324.25), 1.44 (95% UI: 1.14, 1.60), and 150.02 (95% UI: 115.70, 191.80), respectively, indicating that the epidemiological indicators were relatively low compared with the global disease burden. The ASIR, ASPR, ASMR, and ASDR in China were 28.18 (95% UI: 19.30, 37.89), 214.71 (95% UI: 150.10, 278.57), 0.81 (95% UI: 0.68, 1.00), and 101.39 (95% UI: 72.51, 139.41), respectively, indicating that the disease burden was significantly lower than that observed globally and in Asia (Figure 1).

From 1990 to 2021, the epilepsy burden exhibited distinct regional variations globally, in Asia, and in China. Globally, the ASIR increased slightly (EAPC = 0.37), with the largest increase observed in Guinea (EAPC = 1.90 [95% CI: 1.62, 2.18]) and the most significant decline in Burundi (EAPC = −0.97 [95% CI: −1.12, −0.81]). In Asia, the ASIR generally rose slightly, with an EAPC of 0.28 (95% CI: 0.22, 0.33), with the highest increase in Singapore (EAPC 0.47 [95% CI: 0.39, 0.55]), while Democratic People's Republic of Korea showed the steepest decline (EAPC − 0.47 [95% CI: −0.5, −0.44]). China's ASIR remained relatively stable, with an EAPC of 0.12 (95% CI: −0.09, 0.33). The ASPR showed global variations, with Guinea having the largest increase in EAPC of 2.45 (95% CI: 2.04, 2.86) and Burundi the greatest decrease in EAPC of −1.57 (95% CI: −1.79, −1.34). Asia's ASPR experienced a modest rise with an EAPC of 0.09 (95% CI: 0.01, 0.17), while China showed a slight decline with an EAPC of −0.32 (95% CI: −0.56, −0.07), alleviating the burden. Epilepsy ASMR declined in most regions, notably in Estonia, with an EAPC of −3.75 (95% CI: −4.36, −3.13), while increases were observed in some developed countries, such as Italy, with an EAPC of 3.07 (95% CI: 2.77, 3.38). Asia recorded a general decline, with an EAPC of −1.27 (95% CI: −1.35, −1.19), and China exhibited a sharper decrease, with an EAPC of −2.96 (95% CI: −3.09, −2.83), reflecting improved management. The ASDR also showed a global decline, most prominently in Belarus, with an EAPC of −2.55 (95% CI: −2.82, −2.29). Asia experienced a steady reduction, with an EAPC of −1.02 (95% CI: −1.1, −0.95), whereas China demonstrated a marked decline, with an EAPC of −2.38 (95% CI: −2.53, −2.23), indicating a substantial reduction in the overall health burden of epilepsy (Figure 1).

**Figure 2:**
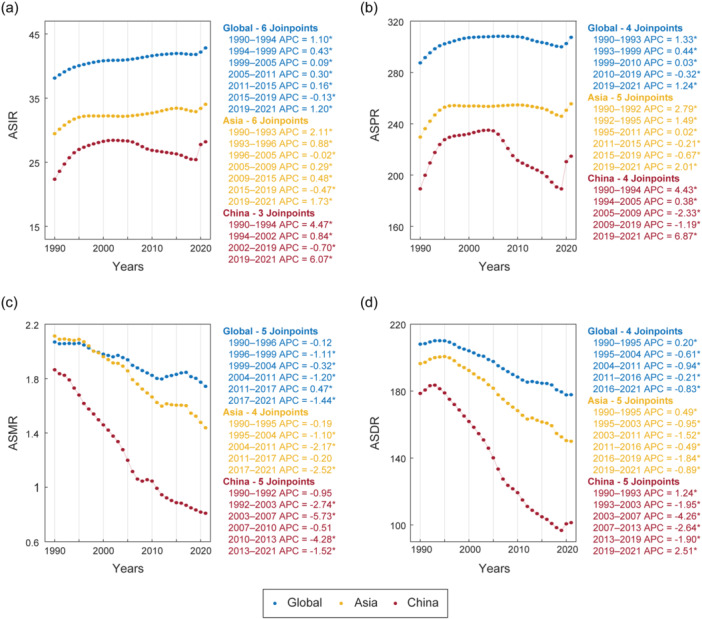
Joinpoint regression analysis of the temporal trend on epilepsy burden globally, in Asia, and in China from 1990 to 2021. (a) ASIR; (b) ASPR; (c) ASMR; and (d) ASDR. *Indicates statistically significant APC (*p* < 0.05). ASDR, age‐standardized disability‐adjusted life years rate; ASIR, age‐standardized incidence rate; ASMR, age‐standardized mortality rate; ASPR, age‐standardized prevalence rate.

The global ASIR showed a slow overall increase, with notable fluctuations. After 2015, the trend reversed from a mild decline (APC = −0.13) to a marked increase (APC = 1.20) after 2019. Asia demonstrated greater variability, with turning points in 1996 (APC = −0.02) and 2015 (APC = −0.47), followed by an accelerated increase in 2019 (APC = 1.73). China exhibited the most dramatic changes, with the ASIR declining from 2002 to 2019 (APC = −0.70) and then sharply rising from 2019 to 2021 (APC = 6.07), exceeding both global and regional trends. The ASPR increased consistently across all regions, especially in China, where it surged between 2019 and 2021 (APC = 6.87). ASMR showed a declining trend globally and in Asia, with China experiencing a rapid drop from 2003 to 2007 (APC = −5.73), followed by a slower decline from 2013 to 2021 (APC = −1.52). The ASDR also declined globally, with China showing the greatest reduction (APC = −4.26 from 2003 to 2007; −1.90 from 2013 to 2019), indicating substantial improvements in disease control (Figure 2).

### 3.2 Health Inequality Analysis

**Figure 3:**
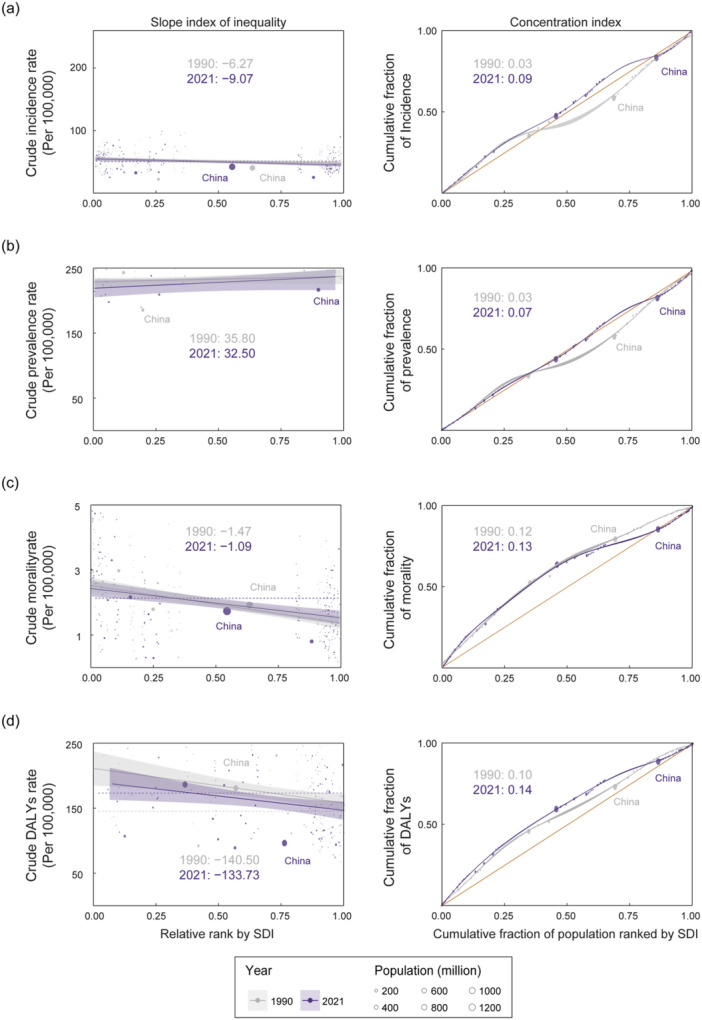
Relationship between health inequality and epilepsy burden in 1990 and 2021. The left panel in each subplot displays the SII regression curves, illustrating the absolute disparities in epilepsy burden across the SDI spectrum. The right panel presents the concentration index curves, reflecting the relative distribution of epilepsy burden by SDI. (a) Incidence, (b) prevalence, (c) mortality, and (d) DALY rate. DALY, disability‐adjusted life years rate; SDI, socio‐demographic index; SII, slope index of inequality.

Globally, the epilepsy burden demonstrates significant health inequalities, with incidence showing widening disparities. The SII decreased from −6.27 in 1990 to −9.07 in 2021, indicating a widening of absolute inequality in epilepsy burden, with the burden becoming more heavily concentrated among individuals with lower socioeconomic status. The concentration index increased from 0.03 to 0.09, reflecting enhanced health inequality. In prevalence, disparities persisted globally, with SII slightly decreasing (35.80 to 32.50) but concentration index rising (0.03 to 0.07), suggesting a continued concentration of cases in high‐SDI countries. For mortality, inequalities showed notable improvements globally, with the SII increasing from −1.47 to −1.09 and the concentration index slightly increasing (0.12 to 0.13). For the DALY rate, absolute socioeconomic inequality slightly improved, as indicated by the increase in SII from −140.50 to −133.73; however, relative inequality increased, with the concentration index rising from 0.10 to 0.14. From both mortality and DALY rate perspectives, the burden of epilepsy was concentrated in populations with higher socioeconomic status, with heightened inequality (Figure 3).

### 3.3 Relationship Between Epilepsy Burden and Social Development

**Figure 4:**
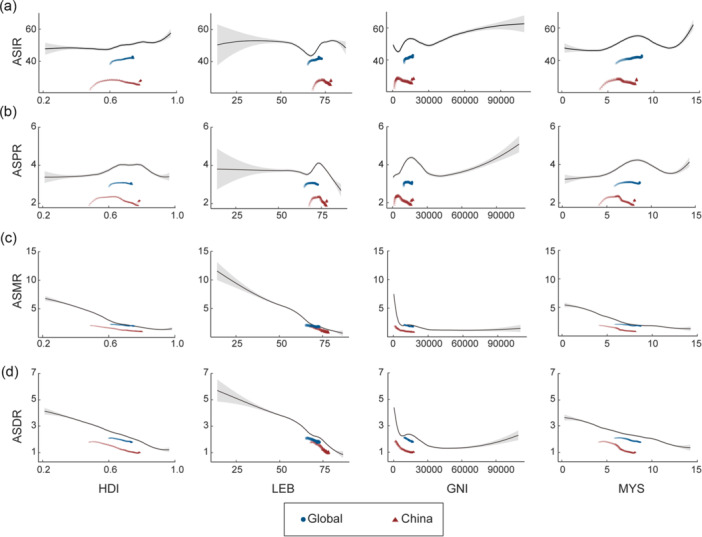
The relationship between epilepsy burden and social development. (a) ASIR; (b) ASPR; (c) ASMR; and (d) ASDR. The black curves represent global nonlinear regression fits, and the gray shaded areas represent their 95% confidence intervals. Blue and red markers represent global and China‐specific estimates, respectively. All rates are expressed per 100,000 population. ASDR, age‐standardized disability‐adjusted life years rate; ASIR, age‐standardized incidence rate; ASMR, age‐standardized mortality rate; ASPR, age‐standardized prevalence rate.

Higher socioeconomic development, as indicated by the HDI, LEB, GNI, and MYS, was consistently associated with a lower epilepsy burden. Globally, countries with higher HDI showed lower ASIR and ASPR, whereas low‐HDI countries experienced a higher burden. In China, rising HDI, GNI, LEB, and MYS were linked to steady declines in ASIR and ASPR, reflecting better disease management. Similar patterns were observed in Asia, although disparities persisted between high‐ and low‐HDI countries. The ASMR decreased globally with higher GNI and education, with China showing the largest reduction. In Asia, countries such as India and Pakistan continue to experience high mortality rates. The ASDR also declined globally, with China demonstrating the most notable improvements, particularly in economically advanced regions (Figure 4).

### 3.4 Relationship Between Epilepsy Burden and Health Service Coverage

**Figure 5:**
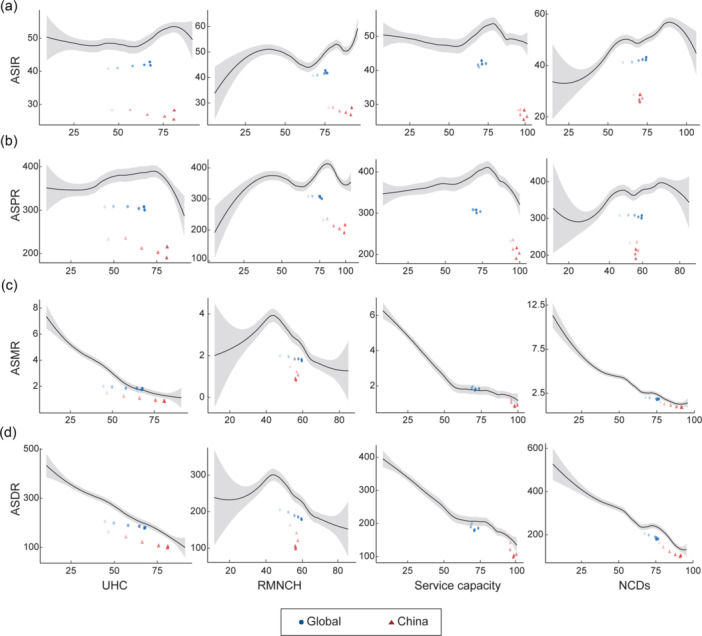
The relationship between epilepsy burden and health service coverage. (a) ASIR; (b) ASPR; (c) ASMR; and (d) ASDR. Blue lines represent global nonlinear trend estimates, and the gray shaded areas represent their 95% confidence intervals. Global and China‐specific data points are plotted in blue and red. All y‐axis values are expressed per 100,000 population. ASDR, age‐standardized disability‐adjusted life years rate; ASIR, age‐standardized incidence rate; ASMR, age‐standardized mortality rate; ASPR, age‐standardized prevalence rate; NCD, non‐communicable disease; RMNCH, reproductive, maternal, newborn, and child health; UHC, universal health coverage.

Higher health service coverage, measured by UHC, RMNCH, service capacity, and NCD management, was associated with a lower epilepsy burden. Globally, countries with high UHC showed lower ASIR, ASPR, ASMR, and ASDR. In China, steady improvements in UHC, RMNCH, service capacity, and NCD management corresponded with a significant decline in the epilepsy burden. Asian trends were consistent with global patterns; however, UHC varied widely among countries. Regions with better chronic disease management systems, particularly in China, experienced the most significant reduction in the epilepsy burden (Figure 5).

### 3.5 APC Analysis of Epilepsy Burden

Globally and in Asia, the incidence of epilepsy exhibits a U‐shaped distribution, with higher rates in childhood and old age. China displayed a similar pattern but with lower rates. The prevalence also followed a U‐shaped curve, peaking in early childhood and among the elderly. Mortality increased exponentially with age, while the DALY rate mirrored this pattern, showing higher values in childhood and old age.

Globally and in Asia, the relative risk of epilepsy incidence in the period effect increased over time, with the global rate ratio rising from 0.96 (95% UI: 0.95, 0.97) in 1990 to 1.06 (95% UI: 1.05, 1.07) in 2021. China showed a unique trend, with an initial decline in the period effect from 1990 to 2000, followed by moderate fluctuations. Prevalence and mortality showed similar trends, with global and Asian values increasing, whereas China demonstrated a decline. For the DALY rate, the period effect decreased globally and in China, reflecting improved disease management.

Globally, early birth cohorts exhibited a higher epilepsy risk, with the relative risk increasing between 1880 and 1950, peaking in 1970 in Asia with a rate ratio of 1.02 (95% UI: 1.01, 1.04) before declining in post‐1980 cohorts. In China, the cohort effect peaked in early birth cohorts but declined significantly after 1980. Mortality was highest in early cohorts globally, with China showing a peak rate ratio of 2.11 (95% UI: 1.74, 2.56) around 1900, followed by a steady decline in mortality. Cohort effects for the DALY rate also declined sharply in the post‐1980 cohorts.

These APC analyses highlighted distinct age, period, and cohort effects on epilepsy burden, with China showing the most significant improvements, particularly in recent decades (Figure S1).

### 3.6 Distribution of Risk Factors for Epilepsy Burden

Globally, epilepsy mortality associated with high alcohol intake showed a slight decline, with the ASMR decreasing from 0.15 (95% UI: 0.11, 0.20) in 1990 to 0.14 (95% UI: 0.10, 0.18) in 2021. Despite this decline, alcohol‐related epilepsy mortality remains high. Notably, the ASMR was consistently higher among men than among women. In 1990, the ASMR for males was 0.27 (95% UI: 0.19, 0.35), which was significantly higher than that for females (0.04 [95% UI: 0.02, 0.05]). By 2021, this gender gap persisted, with men at 0.25 (95% UI: 0.18, 0.33) and women at 0.04 (95% UI: 0.03, 0.06).

Regional differences were also observed. In 1990, the ASMR for all genders in Asia and China was 0.10 (95% UI: 0.07, 0.14) and 0.14 (95% UI: 0.09, 0.20), respectively, both lower than the global average. By 2021, these values had declined further to 0.08 (95% UI: 0.06, 0.11) for Asia and 0.08 (95% UI: 0.05, 0.11) for China, indicating significant progress in reducing alcohol‐related epilepsy mortality (Figure S2a). In terms of ASDR, the global burden of epilepsy for all sexes slightly decreased from 13.74 (95% UI: 9.26, 19.32) in 1990 to 12.51 (95% UI: 8.31, 17.66) in 2021. However, male patients consistently showed a higher ASDR burden than female patients globally. For males, ASDR was 21.55 (95% UI: 15.59, 30.76) in 1990 and 20.94 (95% UI: 14.00, 30.29) in 2021. In Asia and China, the ASDR for all sexes demonstrated more substantial declines than the global average, reaching 13.67 (95% UI: 8.79, 19.53) and 15.35 (95% UI: 9.41, 22.60) in 2021, respectively (Figure S2b).

Alcohol‐related seizures are more common among individuals with chronic alcohol dependence, and men with epilepsy are at a higher risk of death or severe disability due to alcohol‐related complications, including status epilepticus and traumatic brain injury.

### 3.7 Projected Trends of Epilepsy Burden Over the Next 30 Years

Globally, the ASIR of epilepsy is expected to rise over the next 30 years, with the sharpest increase among individuals aged ≥ 80 years. Among women, the ASIR is projected to increase gradually in the older age group (≥ 60 years) while remaining stable among younger individuals (< 40 years old). For men, the projected increase was more pronounced in the older age groups. In Asia, the ASIR is also expected to rise, but at a slower rate than the global average rate. Individuals aged ≥ 60 years will continue to be the main group experiencing an increase in ASIR. In China, the overall ASIR increase will be relatively modest, primarily affecting older adults, especially men aged ≥ 75 years, where the ASIR is projected to reach 21.69 (95% UI: 19.20, 24.17). Among Chinese women aged ≥ 70 years, the ASIR will also increase, reaching 15.59 (95% UI: 13.53, 17.66); however, this growth will be less pronounced than that in men.

ASPR of epilepsy is expected to rise globally, with the largest increase observed among the elderly (≥ 70 years). In children and young adults (< 30 years), ASPR will remain relatively stable. Women will experience a slightly higher ASPR than men, but the difference is minimal. In Asia, ASPR is projected to rise faster than the global average, particularly among the elderly, while in China, the ASPR will show a moderate increase. The largest growth is expected among elderly individuals (≥ 70 years), with ASPR values projected to reach 272.98 (95% UI: 247.46, 306.49) for the general population, 393.80 (95% UI: 357.31, 430.29) for elderly men, and 366.10 (95% UI: 330.33, 401.87) for elderly women.

Globally, ASMR is expected to remain high among the elderly (≥ 70 years), with a projected value of 8.03 (95% UI: 5.31, 10.76) for this age group. In Asia, the decline in ASMR will be slower than the global average, but elderly individuals will still show a projected increase (3.62, 95% UI: 2.71, 4.52). In China, the ASMR will continue to decline overall, but elderly populations will remain at higher risk, with a projected ASMR of 1.06 (95% UI: −0.75, 2.87) among those aged ≥ 70 years. Men are expected to maintain a higher ASMR than women, but the gender gap will gradually narrow.

**Figure 6:**
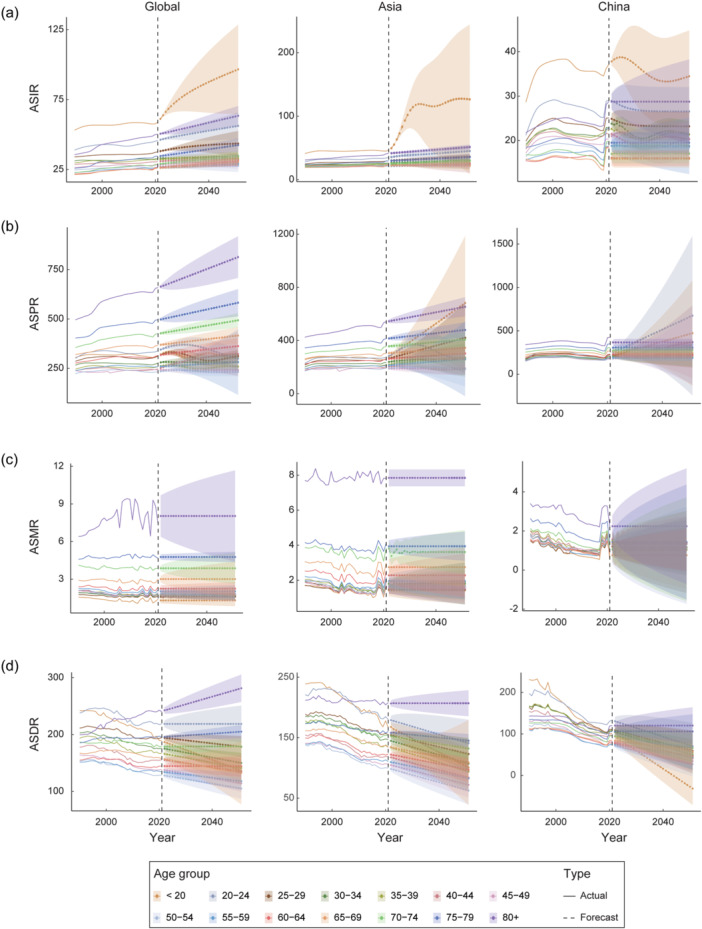
Projected trends of epilepsy burden globally, in Asia and in China over the next 30 years. (a) ASIR; (b) ASPR; (c) ASMR; and (d) ASDR. Different colors represent different 14 age groups: < 20 years old, 20–24, 25–29, 30–34, 35–39, 40–44, 45–49, 50–54, 55–59, 60–64, 65–69, 70–74, 75–79, 80, and above. ASDR, age‐standardized disability‐adjusted life years rate; ASIR, age‐standardized incidence rate; ASMR, age‐standardized mortality rate; ASPR, age‐standardized prevalence rate.

The global ASDR of epilepsy is projected to remain relatively stable after 2020, with significant variations across age groups. Among individuals aged ≥ 70 years, ASDR is expected to increase, reaching 179.08 (95% UI: 169.57, 188.59), while younger age groups (< 40 years) are likely to experience a decline. In Asia, the overall ASDR will decline at a slower rate than the global trend, but older adults (≥ 65 years) will continue to face a substantial burden. China is expected to see a more pronounced decline in ASDR, with overall burden lower than the global and Asian averages, but the elderly population (≥ 65 years) will remain the primary group at risk (Figure 6).

## 4 Discussion

This study provides a comprehensive assessment of global, regional (Asia), and national (China) epilepsy burden trends from 1990 to 2021, with projections to 2050. Using GBD 2021 data and a suite of robust epidemiological models, we identified persistent geographic and temporal disparities in epilepsy outcomes, influenced by sociodemographic and health system factors.

We observed divergent trends in ASIR, ASPR, ASMR, and ASDR. While global ASIR and ASPR modestly increased over three decades, ASMR and ASDR steadily declined. Asia demonstrated faster growth in incidence and prevalence compared to global averages, while China showed the steepest initial increases, followed by notable reductions. China's ASMR declined by an AAPC of −2.64%, reflecting significant health gains. Joinpoint and APC analyses highlighted distinct age and cohort effects, particularly elevated risks among the elderly and early birth cohorts. Furthermore, predictive modeling suggests an ongoing shift of epilepsy burden toward older populations, especially men aged ≥ 70 years.

Our study expands upon prior GBD analyses by providing an updated, granular assessment of epilepsy burden across global, Asian, and Chinese contexts from 1990 to 2021, alongside projections to 2050. Compared to previous GBD iterations, Feigin et al. underscored the continued global rise in epilepsy burden, particularly in low‐ and middle‐income countries [18]. Our findings suggest a more nuanced picture. While incidence and prevalence continue to rise, especially among older populations, mortality and DALYs show a declining trend, likely reflecting improvements in diagnosis, care delivery, and survival. This divergence in burden components echoes findings from Hu et al., who reported persistent gender and socioeconomic disparities, particularly in low‐SDI countries [11]. Our study builds on this by quantifying health inequities using both the SII and the concentration index, offering a clearer depiction of how epilepsy remains concentrated in socially disadvantaged populations. Notably, in Asia and China, we observed marked improvements in both ASMR and ASDR, likely attributable to expanded universal health coverage and treatment advances [19], as reflected by China's UHC index score of 79. Our APC model reveals patterns consistent with prior age‐structured analyses. Shan et al. described the bimodal distribution of epilepsy burden across the lifespan, peaking in childhood and older adulthood [7]. Our results align with this, identifying sharp increases in ASIR and ASPR in those aged ≥ 70, especially in men. This trend highlights the contribution of aging populations and comorbidities, such as stroke, Alzheimer's disease, and traumatic brain injury [20]. In addition to neurological comorbidities, psychiatric conditions, particularly depression and anxiety, are highly prevalent among individuals with epilepsy, with estimated comorbidity rates ranging from 30% to 50% in both clinical and community samples. These psychiatric disorders substantially worsen health‐related quality of life, reduce treatment adherence, and are associated with increased mortality, including suicide. Despite this, current GBD models do not fully capture the additive or synergistic burden posed by these comorbidities on DALYs. As a result, the true global burden of epilepsy may be underestimated, especially in low‐ and middle‐income countries where mental health services are under‐resourced. Future iterations of disease burden estimation should consider incorporating mental health comorbidity‐adjusted disability weights or multimorbidity modeling approaches to better reflect lived experiences and cumulative impairment. Importantly, our study enhances current modeling methodologies. While previous forecasts often relied on linear extrapolation or assumed constant age‐specific rates [21], we applied a multi‐pronged approach: (1) Joinpoint regression to detect temporal inflections; (2) Poisson‐based APC models to disentangle age, period, and cohort effects; and (3) ARIMA time‐series modeling to accommodate nonlinear, stochastic trends. This integrative approach aligns with contemporary best practices in disease forecasting [22, 23]. Compared with analyses from Latin America and sub‐Saharan Africa, our findings highlight that while mortality has improved globally, low‐income regions lag significantly behind in both access and equity. In China, the epilepsy burden is decreasing faster than the regional average, consistent with recent national‐level evaluations [24, 25, 26]. However, sub‐provincial disparities, such as the 1.8‐fold higher DALY rate in western versus eastern China, reflect persistent urban‐rural and regional imbalances, supporting the need for geographically tailored interventions. Taken together, our study confirms, extends, and refines the findings of previous work. It emphasizes that while global trends point toward improving outcomes, these gains are unequally distributed, and older adults, particularly in low‐resource settings, remain a vulnerable group requiring focused intervention. Given the projected increase in epilepsy burden among older adults (≥ 70 years), targeted management strategies are essential. In alignment with the WHO IGAP, we propose integrating epilepsy management into national health policies and UHC to ensure accessibility and affordability [27]. Community‐based screening programs should target high‐risk elderly individuals, especially those with stroke, dementia, or traumatic brain injury, and be integrated into existing rehabilitation pathways. Optimizing antiepileptic drug management, considering polypharmacy and potential interactions, is crucial. Expanding tele‐neurology services will enhance access to specialized care in underserved areas. Promoting brain health and addressing modifiable risk factors (e.g., smoking, alcohol use) are vital for prevention.

Our findings have direct implications for public health strategy, healthcare planning, and policy design. First, the increasing ASIR and ASPR among the elderly underscore the urgent need to integrate epilepsy services into geriatric care frameworks. Given the forecasted rise in ASIR among individuals aged ≥ 80, projected to exceed 28 per 100,000 in Asia and 21 per 100,000 in China, primary care systems must be equipped to recognize and manage epilepsy in aging populations [28]. This includes routine screening for seizures in stroke and dementia clinics and enhanced training for general practitioners in elderly epilepsy management. Second, the clear inverse association between UHC indicators and epilepsy burden reaffirms the importance of expanding equitable access to health services. In China, improvements in maternal and child health services, NCD programs, and medication availability have coincided with a notable reduction in ASMR and ASDR. However, regional disparities persist, particularly in central and western provinces. Policymakers should prioritize resource redistribution through strategies such as mobile epilepsy units, tele‐neurology platforms, and provincial‐level subsidies for antiseizure medications. Third, targeted efforts to reduce alcohol‐related epilepsy risk, especially in men, are essential. Our analysis shows that alcohol‐attributable epilepsy mortality remains disproportionately high in men across all geographies, despite modest global declines in alcohol‐related ASMR from 0.153 to 0.136 per 100,000. Public health campaigns targeting alcohol abuse and injury prevention (e.g., TBI) should explicitly include epilepsy messaging. Evidence‐based programs such as “Screening and Brief Intervention” can be adapted for epilepsy prevention in primary care settings [29]. These gender differences likely reflect a combination of biological susceptibility and social determinants. Higher alcohol consumption among men, often reinforced by cultural norms, contributes to the elevated risk of alcohol‐related seizures, withdrawal, and injury‐related epilepsy. Moreover, men are more frequently employed in high‐risk occupations (e.g., construction, transport), increasing their exposure to traumatic brain injury [30]. Social stigma and lower health‐seeking behavior in men may also delay diagnosis and treatment, further worsening outcomes. Addressing these factors requires gender‐sensitive public health interventions that promote early detection, reduce harmful alcohol use, and improve access to epilepsy care for men. Fourth, persistent socioeconomic disparities necessitate multisectoral approaches. The concentration of burden in low‐SDI countries, reflected in a declining SII and rising concentration index, calls for interagency collaboration between ministries of health, education, and labor. Integrating epilepsy services into broader NCD frameworks, coupled with social protection policies (e.g., epilepsy disability benefits), could reduce treatment gaps and enhance outcomes [31].

In China, policies targeting regional equity, such as strengthening secondary hospitals in underserved areas, training community health workers, and incentivizing neurologist deployment to rural zones, will be critical. Moreover, inclusion of epilepsy in national public health insurance coverage, particularly for long‐term therapy and follow‐up, may reduce financial barriers. Finally, our long‐term projections offer a roadmap for future preparedness. With aging populations driving up epilepsy prevalence and DALYs, health systems must shift toward anticipatory planning. This includes workforce development in neurology and geriatrics, expansion of data surveillance systems (e.g., epilepsy registries), and fostering public‐private partnerships to scale innovative care models. In conclusion, our findings support the WHO IGAP for epilepsy and other neurological disorders (2022–2031), which emphasizes equitable access, integrated care, and life‐course approaches. Implementing IGAP‐aligned strategies, tailored to regional demographic and economic realities, can ensure sustained progress in epilepsy control in the Western Pacific and beyond.

This study benefits from the most current GBD 2021 estimates, longitudinal modeling techniques (e.g., Joinpoint regression, APC, ARIMA), and a focus on Asia and China, where data gaps are prominent. However, limitations include the reliance on modeled estimates rather than primary surveillance data, potential underestimation of cases in resource‐limited settings, and inability to disaggregate epilepsy types (e.g., focal vs generalized). Moreover, projections may be affected by future demographic shifts, healthcare reforms, or emerging risk factors not captured in historical data. Furthermore, the scope of risk factors analyzed is limited to alcohol intake due to data availability. Other important modifiable risk factors, such as smoking, hypertension, traumatic brain injury, and environmental pollution, were not included in our analysis. Future research should explore the impact of these factors on epilepsy incidence and burden.

## 5 Conclusions

Despite notable global progress in reducing epilepsy‐related mortality and DALYs, the incidence and prevalence of epilepsy remain elevated, particularly in low‐SDI settings and among aging populations. Our findings underscore the growing divergence between diagnostic improvements and persistent treatment gaps, especially in resource‐limited regions. Asia, and China in particular, exemplify both the promise and challenge of managing a shifting disease burden, where aging, urbanization, and evolving risk factors intersect. As epilepsy transitions increasingly into a chronic condition in older adults, future strategies must integrate age‐sensitive and equity‐focused care models. Investments in UHC, early diagnosis, access to antiseizure medications, and public health campaigns to reduce modifiable risks, such as alcohol use, will be central to alleviating this burden. Furthermore, addressing the structural inequalities that continue to concentrate the epilepsy burden in low‐income and underserved populations is essential. Projections to 2050 suggest a sustained rise in the absolute number of epilepsy cases, driven primarily by population aging. These forecasts provide a timely foundation for anticipatory policymaking. Targeted interventions at the intersection of neurology, primary care, and social protection will be required to meet the long‐term healthcare needs of people living with epilepsy. Without coordinated action, the global ambition to reduce neurological disease burden may fall short. Our study highlights both the urgency and opportunity to close the epilepsy treatment gap, particularly in the Western Pacific region.

## Author Contributions

**Du Cai:** writing – review and editing. **Xianze Li:** visualization. **Qiwen Yuan:** validation. **Zhongming Lian:** writing – original draft. **Chenyu Zhao:** writing – review and editing, visualization. **Baotian Zhao:** project administration, software. **Xiu Wang:** visualization. **Chao Zhang:** validation, writing – review and editing. **Lin Sang:** visualization, validation. **Wenhan Hu:** writing – review and editing, visualization. **Xiaoqiu Shao:** formal analysis, project administration. **Jianguo Zhang:** validation, visualization. **Shichuo Li:** writing – review and editing, visualization, validation. **Jiajie Mo:** writing – review and editing, visualization. **Kai Zhang:** writing – review and editing, visualization, funding acquisition, conceptualization, supervision, data curation.

## Ethics Statement

All data used in this study are derived from publicly available sources, and none of the data contained in these datasets involve sensitive ethical information. Accordingly, the institutional review board of Beijing Tiantan Hospital has granted an exemption from ethical review.

## Consent

The authors have nothing to report.

## Conflicts of Interest

The authors declare no conflicts of interest.

## Supporting information

Supporting File 1

Supporting File 2

Supporting File 3

## Data Availability

All data and code reported in this article will be shared by the lead contact upon request. Any additional information required to reanalyze the data reported in this article is available from the lead contact upon request. Some of these data presented here are publicly available on the Global Health Data Exchange website, and additional data can be requested from the Institute for Health Metrics and Evaluation.
