# Supplementary material for: Long‐Term Trends and Projections of the Global Epilepsy Burden: Insights From the Global Burden of Disease Study 2021
Source: Health Care Sci. 2026 May 26;5(4):269–83. doi: 10.1002/hcs2.70081 (PMC13398957; doi:10.1002/hcs2.70081)
Supplement: Supplementary file 1 [file HCS2-5-269-s002.docx]

**Supplementary materials**

[Supplementary Methods	2](#_Toc229586179)

[Data source	2](#_Toc229586180)

[Average annual percent change (AAPC)	2](#_Toc229586181)

[Predictive estimation	2](#_Toc229586182)

[Age-period-cohort (APC) model	3](#_Toc229586183)

[Supplementary Results	4](#_Toc229586184)

# Supplementary Methods

## Data source

Disease Modelling Meta-Regression (DisMod-MR) 2.1, a Bayesian meta-regression model, is employed to estimate disease incidence and prevalence. This model takes into account the uncertainty in the data and assigns a standard error to each observation, resulting in more accurate and reliable estimates. This approach allows for consistent and comparable estimates across locations and time periods, as well as enhances the precision of the analysis, particularly when dealing with complex datasets.

## Average annual percent change (AAPC)

Joinpoint regression, a statistical analysis method, is particularly useful for detecting shifts in trends in time series data, such as prevalence, incidence, mortality, and DALYs associated with epilepsy. To analyze these trends, joinpoint regression software can be employed. This software fits a joinpoint model to the data, and employs a Monte Carlo Permutation test to assess the statistical significance of any observed trend alterations, estimating the average annual percent change for each distinct segment and pinpointing the points where the trend undergoes a change. The formula for calculating AAPC is:

$$\text{AAPC}=\left(\frac{\text{e}^{\sum\text{w}_{\text{i}}\text{β}_{\text{i}}}}{\sum\text{w}_{\text{i}}-1}\right)\times\text{100\%}$$

## Predictive estimation

The study used Estimated Annual Percentage Change (EAPC) in order to estimate the trend of age-standardized rates (ASR) in epilepsy, including ASIR, ASPR, ASMR and ASDR. The following formula is used to calculate ASR per 100,000 population.

$$ASR=\frac{\sum_{i=1}^{A} a_{i}\omega_{i}}{\sum_{i=1}^{A} \omega_{i}}\times100,000$$

In this formula, *a_i_* represents the age-specific rate for the *i*-th age group, *ω_i_* indicates the population size of the *i*-th age group in the standardized population, and *A* denotes the total number of age groups. When EAPC and its 95% CI lower bound are both positive, ASR is considered to have an upward trend. Conversely, if both the EAPC and its 95% CI upper limit are negative, ASR is considered to be trending downward. If neither of the two conditions is satisfied, the age normalization rate is considered stable.

## Age-period-cohort (APC) model

According to the Poisson distribution, the APC model was used to analyze the independent effect of age, period and birth cohort on epilepsy burden. In the classical APC model, it is assumed that the epilepsy-related events count follows the Poisson distribution, and the log-linear Poisson model fits the logarithm of the rates.

$$\log(\lambda_{ij})=\mu+\alpha_{i}+\beta_{j}+\gamma_{k}$$

Where *λ_ij_* signifies the data of incidence, prevalence, mortality and DALYs rates of epilepsy, *μ* denotes the mean effect. *α_i_*, *β_j_* and *γ_k_* represent age, period and cohort effects, respectively. The period/cohort effect is expressed by the risk ratio (RR), which is calculated as the age-specific ratio for each period/cohort relative to the reference period/cohort. The APC model building was performed using Stata software (version 14.0, StataCorp LP, Texas, United States).

# Supplementary Results

**Figure S1.** Independent effects of age, period, and cohort effects on epilepsy burden in global, in Asia and China from 1990 to 2021. (a) Incidence; (b) Prevalence; (c) Mortality; (d) Disability-adjusted life years rates.

**Figure S2.** Epilepsy risk projections by high alcohol intake risk factor and sex in global, in Asia and China from 1990 to 2021. (a) ASMR; (b) ASDR.

Abbreviations: ASMR, age-standardized mortality rates; ASDR, age-standardized disability-adjusted life years rates.
