# Supplementary figures and images for: Long‐Term Trends and Projections of the Global Epilepsy Burden: Insights From the Global Burden of Disease Study 2021
Source: Health Care Sci. 2026 May 26:10.1002/hcs2.70081. Online ahead of print. doi: 10.1002/hcs2.70081 (PMC13398957; doi:10.1002/hcs2.70081)

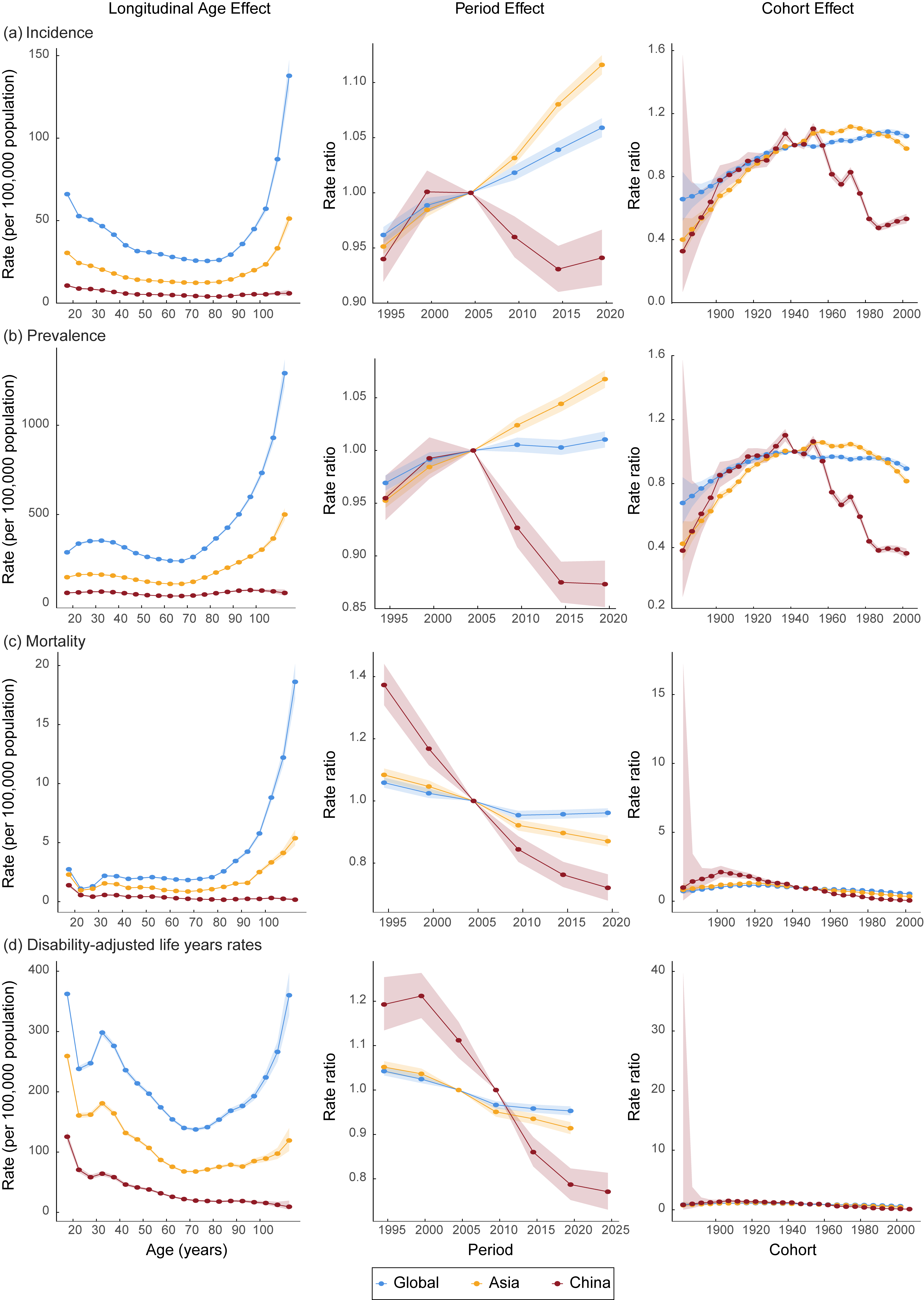

Supplement: Supplementary file 1 — Supporting File 1 [file HCS2-9999-0-s002.tif]

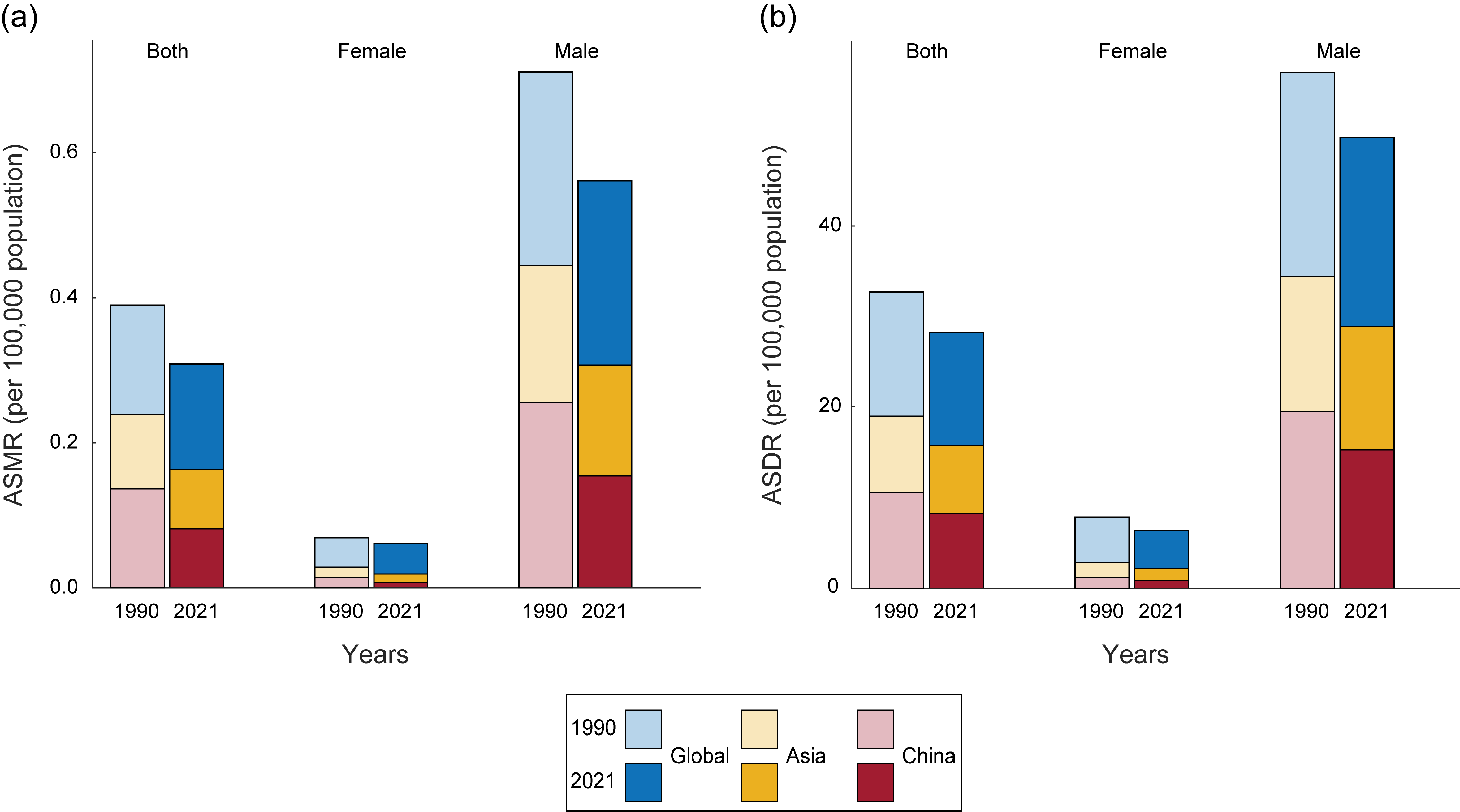

Supplement: Supplementary file 2 — Supporting File 2 [file HCS2-9999-0-s003.tif]
